# Power formulas for mixed effects models with random slope and intercept comparing rate of change across groups

**DOI:** 10.1515/ijb-2020-0107

**Published:** 2021-01-18

**Authors:** Yu Zhao, Steven D. Edland

**Affiliations:** Division of Biostatistics, School of Public Health & Human Longevity Science, University of California San Diego, 9500 Gilman Dr, 92093-0021 La Jolla, USA; Department of Neurosciences, School of Medicine, University of California San Diego, La Jolla, USA

**Keywords:** clinical trial, linear mixed effects model; power, sample size, study subject attrition

## Abstract

We have previously derived power calculation formulas for cohort studies and clinical trials using the longitudinal mixed effects model with random slopes and intercepts to compare rate of change across groups [Ard & Edland, Power calculations for clinical trials in Alzheimer’s disease. J Alzheim Dis 2011;21:369–77]. We here generalize these power formulas to accommodate 1) missing data due to study subject attrition common to longitudinal studies, 2) unequal sample size across groups, and 3) unequal variance parameters across groups. We demonstrate how these formulas can be used to power a future study even when the design of available pilot study data (i.e., number and interval between longitudinal observations) does not match the design of the planned future study. We demonstrate how differences in variance parameters across groups, typically overlooked in power calculations, can have a dramatic effect on statistical power. This is especially relevant to clinical trials, where changes over time in the treatment arm reflect background variability in progression observed in the placebo control arm plus variability in response to treatment, meaning that power calculations based only on the placebo arm covariance structure may be anticonservative. These more general power formulas are a useful resource for understanding the relative influence of these multiple factors on the efficiency of cohort studies and clinical trials, and for designing future trials under the random slopes and intercepts model.

## 1 Introduction

Ref. [1] have previously described sample size formulas for longitudinal studies with study subject dropout for the mixed model repeated measures analysis comparing change from baseline to last visit across groups. Missing data due to study subject dropout in clinical trials and cohort studies is common and reduces statistical power to detect treatment effects or differences in change across groups. We here derive power formulas for longitudinal studies with study subject dropout for a different model, the mixed effects model with random slopes and intercepts comparing mean slope across groups. We demonstrate how power formulas under this model can be used to power a future trial of arbitrary design (arbitrary number and interval between follow-up observation) regardless of the design of pilot study informing power calculations. We expand and generalize previously published mixed effects model power formulas (e.g. [2], [3]) to fully accommodate differences in length and interval between longitudinal observations, different allocation ratios, and different study subject attrition rates. We also derive a formula that accommodates different covariance structures across groups. Differences in covariance are typically ignored, but may be critical to clinical trials, where changes over time in the treatment arm reflect the normal background variability in progression observed in the placebo control arm plus variability in response to treatment, meaning that power calculations based only on the placebo arm covariance structure may be anticonservative. To our knowledge, this is the first presentation of power formulas for the mixed effects model with random slopes and intercepts that accommodates differences in model variance parameters across groups. We note that a substantial literature describes many of these features for mixed model repeated measures analyses assuming compound symmetric or autoregressive covariance of repeated measures [1], [3], [4], [5]. While compound symmetric and autoregressive covariance structures are mathematically more tractable, in our experience these models are not appropriate for repeated measures of chronic progressive conditions. We demonstrate by example that compound symmetric and autoregressive covariance structures typically are not appropriate for modeling chronic progressive conditions. In the interest of clarity, in this paper we focus exclusively on the model with covariance structure imposed by random slopes and intercepts most appropriate for chronic progressive outcome measures.

## 2 Background, the mixed effects model

The parameterization of the mixed effects model with random slopes and intercepts used in this derivation is the familiar Laird and Ware mixed effects model parameterization with estimation and hypothesis testing by restricted maximum likelihood (REML). We use the notation of [6] to represent within group longitudinal observations $y_{i}$ on subject *i* as

(1) $$y_{i}=X_{i}\alpha +Z_{i}b_{i}+e_{i}$$

where α are the fixed effect intercept and slope describing the mean longitudinal trajectory, $b_{i}∼N\left(0,D\right)$ are random, subject-specific intercepts and slopes, and $e_{i}∼N\left(0,R_{i}\right)$ is residual variation about the individual trajectories. When convenient, we will represent the elements of D as $\left(\begin{array}{ll} \sigma_{b_{0}}^{2} & \sigma_{b_{0},b_{1}}\\ \sigma_{b_{0},b_{1}} & \sigma_{b_{1}}^{2} \end{array}\right)$ . In the derivation below, $X_{i}=Z_{i}$ are subject specific design matrices composed of a column of ones and a column of times at which measurements $y_{i}$ were made. To simplify presentation we maintain large sample normality assumptions in all that follows, and we do not consider covariates beyond $t_{i}$ . Consistent with prior literature [2], [3], we assume that data are missing at random and that the covariance parameters are known.

Ref. [7] showed that $V\left(\hat{\alpha }\right)$ , the asymptotic variance of maximum likelihood estimates of α , is independent of $\hat{\alpha }$ and derived its value. Under model (1), *y* is normally distributed with mean Xα and variance-covariance V . The likelihood function is

(2) $$l={\left(2\pi \right)}^{-\frac{1}{2}n}{\left|V\right|}^{\frac{1}{2}}\;\exp\left(-\frac{1}{2}{\left(y-X\alpha \right)}^{\prime }V^{-1}\left(y-X\alpha \right)\right)$$

The log likelihood, apart from a constant is

(3) $$L=-\frac{1}{2}\log\left|V\right|-\frac{1}{2}{\left(y-X\alpha \right)}^{\prime }V^{-1}\left(y-X\alpha \right)$$

By the $\sqrt{n}$ -consistency and asymptotic efficiency of MLE, $\hat{\alpha }$ the maximum likelihood estimate of *α* follows

(4) $$\sqrt{n}\left(\hat{\alpha }-\alpha \right)\overset{d}{\to }N\left(0,I^{-1}\left(\alpha \right)\right)$$

where I(α) is the information matrix which equals to $E\left(\frac{\partial^{2}L}{\partial \alpha_{h}\partial \alpha_{k}}\right)$ . For the log likelihood (3), after taking the partial derivative and expectation,

(5) $$I\left(\alpha \right)=X^{\prime }V^{-1}X$$

Thus the asymptotic variance of $\hat{\alpha }$ is

(6) $$V\left(\hat{\alpha }\right)={\left(X^{\prime }V^{-1}X\right)}^{-1}$$

We can further simplify this as

(7) $$\begin{array}{c} V\left(\hat{\alpha }\right)={\left(X^{\prime }V^{-1}X\right)}^{-1}\\ ={\left(\left(X_{1}^{\prime },X_{2}^{\prime },\ldots ,X_{n}^{\prime }\right)\left(\begin{array}{llll} V_{1}^{-1} & 0 & 0 & 0\\ 0 & V_{2}^{-1} & 0 & 0\\ 0 & 0 & \ddots & 0\\ 0 & 0 & 0 & V_{n}^{-1} \end{array}\right)\left(\begin{array}{l} X_{1}\\ X_{2}\\ \vdots \\ X_{n} \end{array}\right)\right)}^{-1}\\ ={\left(\sum_{i}\left(X_{i}^{\prime }V_{i}^{-1}X_{i}\right)\right)}^{-1} \end{array}$$

where

(8) $$V_{i}=\text{Cov}\left(y_{i}\right)=Z_{i}DZ_{i}^{\prime }+R_{i}$$

In particular, the lower right diagonal of $V\left(\hat{\alpha }\right)$ is the variance of the mean slope estimate which is required for sample size formulas to power clinical trials comparing mean slope in treatment versus control. The components of $V\left(\hat{\alpha }\right)$ can be estimated by REML [6].

Two specific cases of Eq. (7) are useful for illustrative purposes. If we are dealing with balanced data, then $X_{i}$ and $V_{i}$ are constant across subjects, and Eq. (7) reduces to simply

(9) $$V\left(\hat{\alpha }\right)={\left(nX_{i}^{\prime }V_{i}^{-1}X_{i}\right)}^{-1}$$

A similar clinical trial with missing observations due to missed clinical exams or study subject dropout would not have constant $V_{i}$ and $X_{i}$ , but instead would have a finite set of design and variance matrix pairs. Letting *k* index this set, the variance of the fixed effect estimates for a clinical trial with missing data is then equal to

(10) $$V\left(\hat{\alpha }\right)={\left(\sum_{k}n_{k}\left(X_{k}^{\prime }V_{k}^{-1}X_{k}\right)\right)}^{-1}={\left(n\sum_{k}p_{k}\left(X_{k}^{\prime }V_{k}^{-1}X_{k}\right)\right)}^{-1}$$

where the $n_{k}$ are counts of subjects in each set and sum to *n*, and $p_{k}=n_{k}/n$ .

## 3 Power formulas derived

### 3.1 Power formula, balanced design with no dropout

For the balanced design with no dropout, standard power formulas apply. E.g., for equal allocation to arms, sample size to detect a difference in mean slope Δ between treatment and control is

(11) $$N/Arm=2{\left(z_{\alpha /2}+z_{\beta }\right)}^{2}{\left[{\left(X_{i}^{\prime }V_{i}^{-1}X_{i}\right)}^{-1}\right]}_{22}/\Delta^{2}$$

This formula can be used given an estimate of $V_{i}=\text{Cov}\left(y_{i}\right)$ obtained from pilot data or a previously completed trial of comparable design.

A more generally applicable formula can be derived given the usual assumption of independent residual error $\left(R_{i}=\sigma_{\epsilon }^{2}I\right)$ . Under this assumption, it can be shown (Appendix A) that ${\left[{\left(X_{i}^{\prime }V_{i}^{-1}X_{i}\right)}^{-1}\right]}_{22}=\left(\sigma_{b_{1}}^{2}+\sigma_{\epsilon }^{2}/\sum {\left(t_{j}-\bar{t}\right)}^{2}\right)$ [8], and Eq. (11) reduces to

(12) $$N/Arm=2{\left(z_{\alpha /2}+z_{\beta }\right)}^{2}\left(\sigma_{b_{1}}^{2}+\sigma_{\epsilon }^{2}/\sum {\left(t_{j}-\bar{t}\right)}^{2}\right))/\Delta^{2}$$

where $\Sigma {\left(t_{j}-\bar{t}\right)}^{2}$ is the sum over the measurement time vector $t={\left(t_{1},t_{2},\ldots .,t_{m}\right)}^{\prime }$ of the squared differences $t_{j}$ minus mean time.

Equation (12) is more generally applicable because it only requires estimates of $\sigma_{\epsilon }^{2}$ and $\sigma_{b_{1}}^{2}$ , which can be obtained by REML fit to longitudinal pilot data of arbitrary design. That is, future studies can be powered using prior study data that do not necessarily have the same duration or interval between follow-up as the planned future study [9]. Equation (12) also provides a heuristic illustration of the influence of study design on power – longer trials or trials with more longitudinal observations increase power by reducing the influence of $\sigma_{\epsilon }^{2}$ on overall variance.

### 3.2 Power formula, balanced design with dropout

Another important example, following Lu et al., is the case of study subject dropout during a cohort study or clinical trial, also referred to as study subject attrition (SSA). SSA implies a subset of the dropout patterns indexed by *k* in Eq. (10), restricting to the m−1 longitudinal dropout patterns composed of subjects whose last visit is at $t_{k}$ , *k* = 2 through *m* inclusive. Given the independent residual errors assumption and equal allocation to arms, under SSA the sample size is calculated by

(13) $$N/Arm=2{\left(z_{\alpha /2}+z_{\beta }\right)}^{2}\left({\left[{\left(\Sigma p_{k}\left(X_{k}^{\prime }V_{k}^{-1}X_{k}\right)\right)}^{-1}\right]}_{22}/\Delta^{2}\right)$$

where the sum is over the m−1 dropout patterns defined by SSA, $p_{k}\left(X_{k}^{\prime }V_{k}^{-1}X_{k}\right)$ are as in Eq. (10), and $V_{k}$ are matrices with off diagonal elements u,v equal to $\sigma_{b_{0}}^{2}+\left(t_{u}+t_{v}\right)\sigma_{b_{0},b_{1}}+t_{u}t_{v}\sigma_{b_{1}}^{2}$ and diagonal elements u,u equal to $\sigma_{b_{0}}^{2}+2t_{u}\sigma_{b_{0},b_{1}}+t_{u}^{2}\sigma_{b_{1}}^{2}+\sigma_{\epsilon }^{2}$ . As before, the parameters $\sigma_{b_{0}}^{2}$ , $\sigma_{b_{0},b_{1}}$ , and $\sigma_{b_{1}}^{2}$ of D and the residual error variance $\sigma_{\epsilon }^{2}$ are estimated by REML fit to representative prior longitudinal data.

Power formulas accommodating study subject attrition such as Eq. (13) and [1] are technically anticonservative because they ignore information lost by the occasional missed interim visit, although this bias is typically small. If missing interim visit data is a concern, then applying Eq. (13) over all sets of missing data patterns will ensure true nominal type I error rates are maintained.

### 3.3 Power formula, unequal allocation, unequal study subject attrition, and unequal variance across groups

Formulas (12) through (13) assume that variance parameters and study subject attrition rates are the same in the two groups being compared and the number of subjects in each group is equal. We may require a formula that accommodates different study subject attrition rates across groups, and/or unequal allocation to groups [1]. It would also be useful to have a formula that accommodates different variance parameters across groups. Letting $Term_{1}$ and $Term_{2}$ indicate the values ${\left[{\left(\Sigma p_{k}\left(X_{k}^{\prime }V_{k}^{-1}X_{k}\right)\right)}^{-1}\right]}_{22}$ calculated separately for $group_{1}$ and $group_{2}$ , and given the independent identically distributed residual error assumption, sample size for $group_{1}$ can be calculated by

(14) $$N_{group_{1}}={\left(z_{\alpha /2}+z_{\beta }\right)}^{2}\left(Term_{1}+\lambda Term_{2}\right)/\Delta^{2})$$

where *λ* is the sample size ratio across groups $\left(N_{group_{2}}=N_{group_{1}}/\lambda \right)$ . The derivation of Eq. (14) is straightforward, and follows from the observation that the variance of the difference in fixed effects slope estimates equals the sum of the individual slope estimate variances. Factoring out $1/N_{group_{1}}$ from this sum leaves the quantity $\left(Term_{1}+\lambda Term_{2}\right)$ , and power as a function of $N_{group_{1}}$ follows.

### 3.4 Modeling under the unequal variance across groups assumption

It is given that using Eq. (14) with unequal variance parameters to power a study presumes the analysis plan for the study explicitly models the covariance structure of the two groups. For most applications, including clinical trials, $\sigma_{\epsilon }^{2}$ is assumed constant across groups. Sample syntax explicitly modeling the remaining, within group random effects parameters determining the covariance structure of repeated measures is included in Appendix B.

## 4 Example

Given representative pilot data it is a simple matter to estimate the variance terms required for the power formulas. For example, Table 1 is the output from a mixed effect model fit to longitudinal ADAS-cog scores observed in the ADCS trial of a folic acid/B6/B12 compound to slow the progression of Alzheimer’s disease [10] (*n* = 330 subjects and *m* = 7 observations per subject) using the software provided with the standard mixed effects model text *Mixed-Effects Models in S and S-PLUS* [11]. The correlation of repeated measures estimated by the random slopes and random intercepts REML model fit (Table 2) mirrors the empirical correlation calculated from the same sample data, confirming that this model well represents the covariance structure of longitudinal repeated measures of a chronic progressive condition. In contrast, the commonly assumed compound symmetric and autoregressive covariance structures are constant on the diagonals and inconsistent with these longitudinal data of a chronic progressive condition.

**Table 1:** Sample model fit using the R package nlme and R function lme.

lme(*y* ^∼^ time, random = ^∼^ time|id)
Random effects formula: ^∼^time | subject
StdDev Corr
(Intercept) 7.432548 (Intr)
Time 3.964215 0.465
residual 3.705466
Fixed effects: ADAS ^∼^ time
Value Std.Error
(Intercept) 17.745024 0.4321112
Time 4.057879 0.2672020
Number of observations: 2310/Number of groups: 330

**Table 2:** Correlation matrices estimated using data from the ADCS Folate/B6/B12 clinical trial. The correlation matrix imposed by a random effect model fit (RE, bottom panel) closely mirrors the empirical correlation matrix (top panel).

Empirical correlation matrix
$\left(\begin{array}{lllllll} 1 & 0.83 & 0.82 & 0.79 & 0.80 & 0.80 & 0.76\\ 0.83 & 1 & 0.84 & 0.82 & 0.82 & 0.85 & 0.80\\ 0.82 & 0.84 & 1 & 0.85 & 0.85 & 0.85 & 0.84\\ 0.79 & 0.82 & 0.85 & 1 & 0.88 & 0.85 & 0.84\\ 0.80 & 0.82 & 0.85 & 0.88 & 1 & 0.90 & 0.88\\ 0.80 & 0.85 & 0.85 & 0.85 & 0.90 & 1 & 0.89\\ 0.76 & 0.80 & 0.84 & 0.84 & 0.88 & 0.89 & 1 \end{array}\right)$

Correlation matrix estimated assuming RE
$\left(\begin{array}{lllllll} 1 & 0.81 & 0.80 & 0.80 & 0.78 & 0.77 & 0.76\\ 0.81 & 1 & 0.83 & 0.83 & 0.82 & 0.81 & 0.81\\ 0.80 & 0.83 & 1 & 0.85 & 0.85 & 0.85 & 0.84\\ 0.80 & 0.83 & 0.85 & 1 & 0.87 & 0.87 & 0.87\\ 0.78 & 0.82 & 0.85 & 0.87 & 1 & 0.88 & 0.89\\ 0.77 & 0.81 & 0.85 & 0.87 & 0.88 & 1 & 0.90\\ 0.76 & 0.81 & 0.84 & 0.87 & 0.89 & 0.90 & 1 \end{array}\right)$

From Table 1, the estimated standard deviation of slopes ${\hat{\sigma }}_{b_{1}}$ is 3.964 and the estimated standard deviation of residual errors ${\hat{\sigma }}_{\epsilon }$ is 3.705 (Table 1). Assuming equal variance across arms, and using these values in Eq. (12), the sample size required to detect a 25% slowing of cognitive decline (Δ=0.25*4.06) with 80% power and a type I error rate of 5% for an 18 month trial with observations every three months is 360 subjects/arm. For comparison, a 24 month trial with observations every three months would require 296 subjects per arm using Eq. (12). Note that it is not necessary for the design of the pilot study (i.e., the number of observations and interval between observations) to match the design of the future trial, we only require that there are sufficient pilot data to estimate the variance parameters $\sigma_{b_{1}}^{2}$ and $\sigma_{\epsilon }^{2}$ .

## 5 Validation by computer simulation

**Figure 1:**
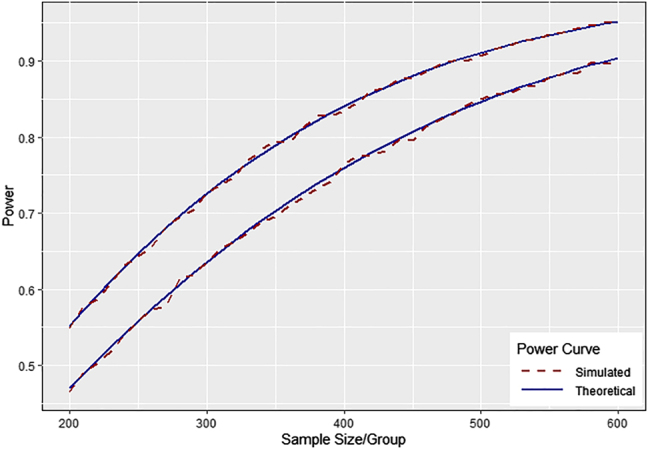
Theoretical power curves versus power estimated by computer simulation given no study subject attrition (top curve) and give 5% attrition per follow-up visit (bottom curve) (10,000 simulations per sample size, two-sided test, type I error α=0.05 ).

To evaluate the performance of Eq. (12) through (14) we have performed computer simulations assuming data following the model fit obtained in the Example above. We first performed simulations assuming a clinical trial with balanced design with six post-baseline time points with no loss to follow-up and with equal variance within arms consistent with Eq. (12). Simulating a series of clinical trials with sample size from 200 to 600 subjects per arm with effect size equal to a 25% reduction in the mean rate of decline observed in placebo (25% of the mean 4.06 points per year rate of decline observed in the pilot data (Table 1)) with 10,000 simulations per sample size simulated, we found that simulated power closely tracks the power predicted by Eq. (12) (top line, Figure 1).

To validate the power formula for data with study subject attrition described in Eq. (13), we simulated data under equivalent conditions, except that for each simulation we randomly dropped 5% of the initial sample from each arm at $t_{2}$ through $t_{7}$ . We similarly found that simulated power closely tracks the power predicted by Eq. (13) power formula (bottom line, Figure 1). Study power decreases when there is study subject attrition (Figure 1).

**Figure 2:**
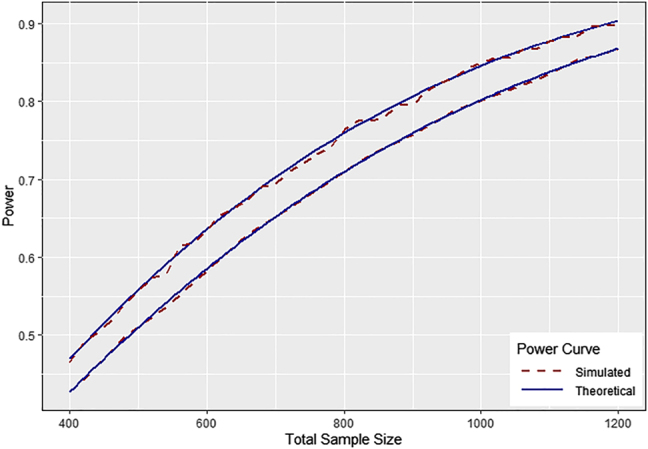
Theoretical powers curve versus power estimated by computer simulation given 5% study subject attrition per visit, and allocation ratio λ=1 (top curve) and λ=2 (bottom curve) (10,000 simulations per sample size, two-sided test, type I error α=0.05 ).

To validate the power formula for data with unequal allocation to groups described in Eq. (14), we simulated data with 5% study subject attrition at each follow-up visit as above, but let the allocation ratio *λ* vary from one to two. Simulated power closely tracks the power predicted by Eq. (14) power formula (Figure 2). Predictably [12], [13], power is maximized when *λ* equals one, and declines as the allocation ratio deviates from one (Figure 2).

**Figure 3:**
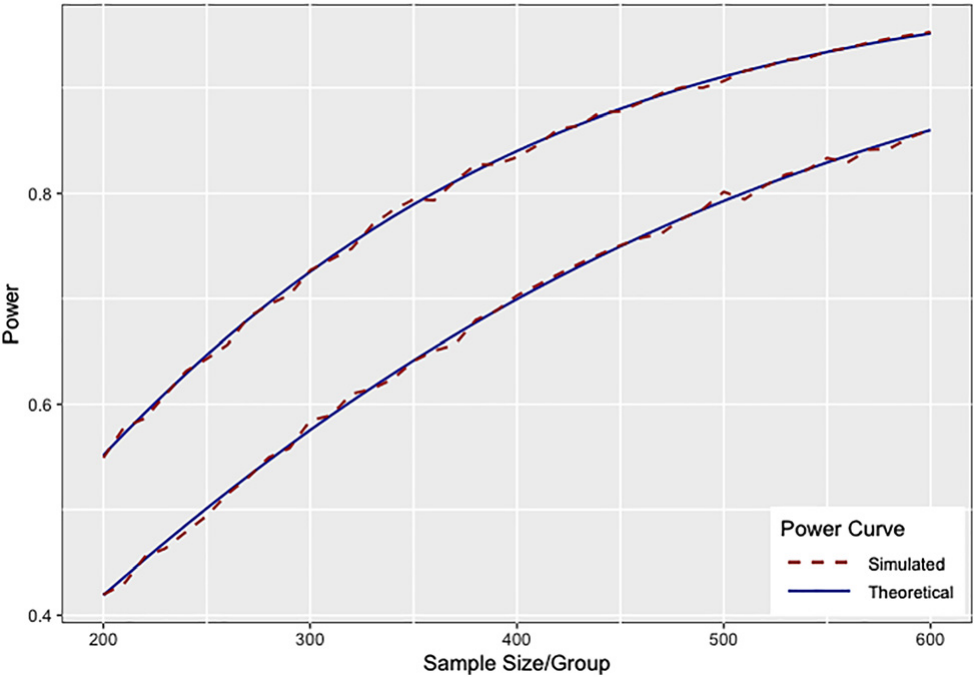
Theoretical power curves versus power estimated by computer simulation given equal variance of random slopes (top line) and given $\sigma_{b_{1}}$ is increased by 50% in one of the groups (bottom line) (10,000 simulations per sample size, two-sided test, type I error α=0.05 ).

To validate Eq. (14) power formula when covariance structures differ across groups, we simulated data as done in the top line of Figure 1, but increased $\sigma_{b_{1}}$ by 50% in one of the groups. Simulated power closely tracks the power predicted by Eq. (14) power formula (Figure 3). The top line from Figure 1 is included in Figure 3 for reference. Figure 3 illustrates the potential for anticonservative power calculations in the clinical trial setting when variance parameters used in power calculations are informed by prior placebo arm data and assumed to be constant across arms.

## 6 Discussion

There are limitations to the Laird and Ware model as parameterized in Eq. (1), because this model depends on the assumption that mean trajectories are linear as a function of time. This assumption may be violated, particularly in clinical trials of treatments with potential acute treatments effect beyond simple alteration of rate of progress of disease. In this circumstance mixed model repeated measure analysis [1] or model robust alternatives such as generalized estimating equations [14] would be preferred. In our experience the linearity assumption is often appropriate for chronic progression conditions, especially when the interval of observation under study is small relative to the full trajectory of disease.

We further note that the formulas presented here assume variance parameters are known, as is typical of the power formula literature [1], [2], [3], [5], [15]. However, variance parameters may be uncertain if sample size in pilot studies used to estimate the variance parameters is small or if pilot data are not perfectly representative of the future investigation being powered. There is a literature on characterizing power when variance parameter estimates are uncertain (e.g. [16]). However, these methods apply to narrow applications that do not include random effects models. We recommend sensitivity analyses using a range of plausible variance parameters to ensure that planned future investigations are adequately powered. If the prior data informing power calculations are available, sensitivity analyses may be informed by bootstrap estimates of the uncertainty of variance parameter estimates (e.g., [17]). We have also used computer simulations to explore the adequacy of pilot study sample size to inform future trials in other applications [18].

The formulas derived here are useful for determining the relative efficiency of different study designs using the mixed effects model to test for differences in mean rate of change between groups. We have described how efficiency can vary by the number and interval between observations, the study subject attrition rate, the allocation ratio, and by differences in variance parameters between groups. Increasing the length of observation or number of observations increases statistical power, although with diminishing returns depending on the magnitude of residual error variance of the outcome measure under study (see Eq. (12)). Study subject attrition can also meaningfully impact statistical power and should be accounted for in study design (see Eq. (13) and, e.g., Figure 1).

Regarding recruitment allocation ratios, if all other conditions are equal across groups, then altering the allocation ratio from one-to-one reduces statistical power for given study sample size [12]. Altering the allocation ratio has been propose to improve statistical power when there is differential attrition rates across clinical trial arms [1]. More commonly, allocation ratios are altered to increase the probability of randomization to the active treatment in the hope of increasing clinical trial recruitment rates. While this approach may increase recruitment rates, it also implies more subjects will have to be recruited to achieve target statistical power, and trade-offs between clinic trial cost and time to completion should be considered carefully when planning a trial with unequal randomization to arms [13].

Finally, we describe how statistical power depends on variance parameters which may vary across groups (Eq. (14)). This consideration is typically overlooked, but may be especially relevant to clinical trials, where rate of progression in the active treatment arm is a function of both underlying variability in rate of progression and variability in response to treatment. Given that response to treatment is unlikely to be constant across subjects, we can anticipate that the variance of random slopes in the treatment arm will be larger than variance in the control arm if there is a treatment effect. Hence, power calculations based only on the covariance within placebo data will be anticonservative. Typically pilot data for clinical trials are from placebo arm data of a previous trial or registry trial with no treatment arm. A conservative power calculation assumption under these circumstances would be to use an inflation factor for $\sigma_{b_{1}}^{2}$ within the treatment arm in (14) to be more likely to achieve nominal power in the planned trial.

Formulas (12), (13), and (14) are implemented in the R package longpower [19], and will be useful tools for planning future cohort studies and clinical trials as well as for comparing the influence of the many factors affecting the efficiency of such investigations. Areas of additional research include modifying power calculation methods in anticipation of evolving guidelines on statistical analysis plans for clinical trials in the presence of missing not at random data [20], and generalizing power formulas to more directly address the stochastic nature of covariance parameter estimates typically used in practice.

## Appendix A

To derive the variance term in Eq. (12), we need to find the bottom right corner of ${\left(X_{i}^{\prime }V_{i}^{-1}X_{i}\right)}^{-1}$ . As derived by [21],

$$\begin{array}{c} V_{i}^{-1}={\left(X_{i}DX_{i}^{T}+\sigma_{\epsilon }^{2}I\right)}^{-1}\\ =\sigma_{\epsilon }^{-2}I-\sigma_{\epsilon }^{-2}X_{i}{\left(X_{i}^{\prime }X_{i}\right)}^{-1}X_{i}^{\prime }+X_{i}{\left(X_{i}^{\prime }X_{i}\right)}^{-1}{\left(\sigma_{\epsilon }^{2}{\left(X_{i}^{\prime }X_{i}\right)}^{-1}+D\right)}^{-1}{\left(X_{i}^{\prime }X_{i}\right)}^{-1}X_{i}^{\prime } \end{array}$$

Substituting and collecting terms,

$${\left(X_{i}^{\prime }V_{i}^{-1}X_{i}\right)}^{-1}=\sigma_{\epsilon }^{2}{\left(X_{i}^{\prime }X_{i}\right)}^{-1}+D$$

and

$${\left[{\left(X_{i}^{\prime }V_{i}^{-1}X_{i}\right)}^{-1}\right]}_{22}=\sigma_{\epsilon }^{2}/\sum {\left(t_{j}-\bar{t}\right)}^{2}+\sigma_{b_{1}}^{2}$$

## Appendix B

The random effects model with random slopes and intercepts can be performed with the lmer function within the R package lmerTest [22]. To test for differences in slopes between groups under the assumption of equal covariance structure in the two groups, the lmer model call is

lmer (Y ^∼^ GROUP ∗ TIME + (TIME | ID))

where ID indexes individual subjects, GROUP is a 0, 1 variable indicating placebo (0) and active treatment (1), and TIME are times of repeated observations on the dependent variable *Y*.

To test for differences in slopes between groups under the assumption of unequal covariance structure in the two groups, as implemented in power Formula (14), the lmer model call is

TIME_0 <- ifelse (GROUP = = 0, TIME, 0)

TIME_1 <- ifelse (GROUP = = 1, TIME, 0)

lmer (Y ^∼^ TIME * GROUP + (TIME_0|ID) + (TIME_1|ID))
